# Rare-earth metal catalysts for high-pressure synthesis of rare diamonds

**DOI:** 10.1038/s41598-021-88038-5

**Published:** 2021-04-19

**Authors:** Yuri N. Palyanov, Yuri M. Borzdov, Igor N. Kupriyanov, Alexander F. Khohkhryakov, Denis V. Nechaev

**Affiliations:** 1 V.S. Sobolev Institute of Geology and Mineralogy Siberian Branch of the Russian Academy of Sciences, Academican Koptyug Ave., 3, Novosibirsk, 630090 Russian Federation; 2 Novosibirsk State University, Pirogova Str., 2, Novosibirsk, 630090 Russian Federation

**Keywords:** Materials science, Physics

## Abstract

The combination of the unique properties of diamond and the prospects for its high-technology applications urges the search for new solvents–catalysts for the synthesis of diamonds with rare and unusual properties. Here we report the synthesis of diamond from melts of 15 rare-earth metals (REM) at 7.8 GPa and 1800–2100 °C. The boundary conditions for diamond crystallization and the optimal parameters for single crystal diamond synthesis are determined. Depending on the REM catalyst, diamond crystallizes in the form of cube–octahedrons, octahedrons and specific crystals bound by tetragon–trioctahedron and trigon–trioctahedron faces. The synthesized diamonds are nitrogen-free and belong to the rare type II, indicating that the rare-earth metals act as both solvent–catalysts and nitrogen getters. It is found that the REM catalysts enable synthesis of diamond doped with group IV elements with formation of impurity–vacancy color centers, promising for the emerging quantum technologies. Our study demonstrates a new field of application of rare-earth metals.

## Introduction

Traditional solvents–catalysts for diamond synthesis at high-pressure high-temperature conditions (HPHT) are group VIII transition metals, particularly Fe, Co, Ni, and their alloys^1–3^. The processes of diamond crystallization in these systems have been thoroughly investigated and form the basis for the modern technologies for the production of synthetic diamond abrasive powders and the growth of large high-quality single crystals^4–6^. In recent decades, studies have been actively carried out on diamond crystallization in various metallic and non-metallic solvent–catalysts, among which elemental substances have turned out very promising. For example, synthetic n-type semiconducting diamonds^7^ and superconducting diamonds^8^ were produced in binary phosphorus–carbon and boron–carbon systems, respectively. At present, special interest in diamond is determined by the possibility of creating in its lattice optically and magnetically active centers^9–11^. Under HPHT conditions, diamonds with germanium-vacancy centers were synthesized in the Ge–C system^12^.

Of particular interest are the catalysts that create ultra-reducing conditions for diamond synthesis and possess nitrogen getter properties. Such properties are characteristic of magnesium^13^. It has been experimentally demonstrated that the diamond growth systems based on Mg catalysts provide conditions for the incorporation into the diamond lattice of group IV elements such as silicon^14^, germanium^15^, and even tin^16^ with the formation of silicon-vacancy (SiV), germanium-vacancy (GeV) and tin-vacancy (SnV) color centers, which are of interest for the emerging quantum technologies^17–19^. Thus, the search for new catalysts for the synthesis of diamond appears to be very urgent. As new, potentially promising media for the synthesis of rare and unusual diamonds, in this work we have experimentally tested different rare earth metals, which have practically not been studied as solvents–catalysts for diamond synthesis. To date, there is only one work reporting on the synthesis of polycrystalline diamond at HPHT conditions in the Eu–C, Tm–C, and Er–C systems^20^.

## Results and discussion

### Diamond synthesis in the REM-C systems

**Figure 1:**
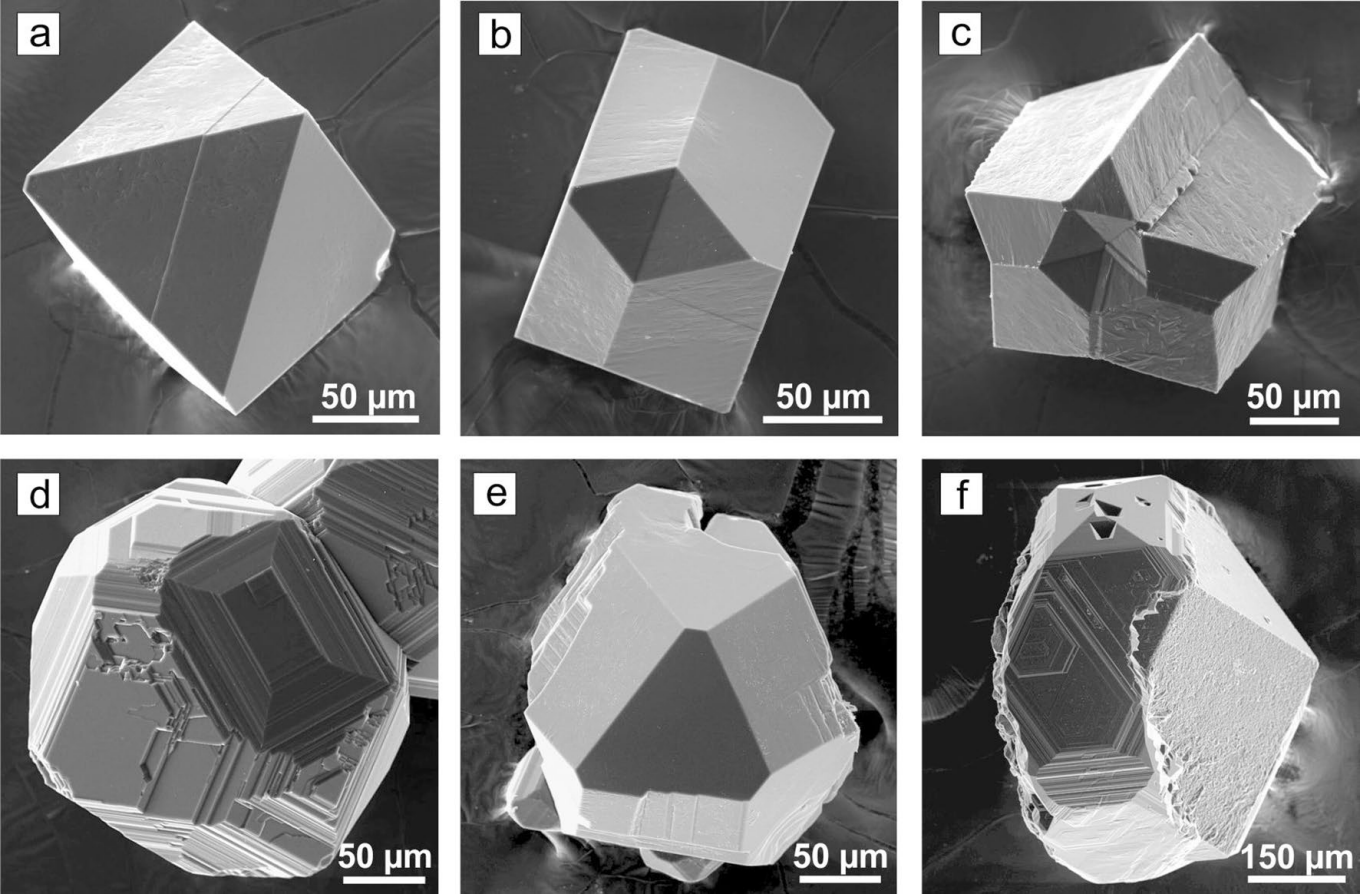
SEM micrographs of diamond crystals synthesized in REM-C systems. (**a**) An octahedral crystal, La–C system; (**b**) a contact twin of octahedral crystals, Ce–C system; (**c**) a cyclic twin of octahedral crystals, Ce–C system; (**d**) crystal of cuboctahedral habit with additional faces, Gd–C systems; (**e**) cuboctahedral crystals, Dy–C system; (**f**) diamond grown on a seed, Ho–C system.

**Figure 2:**
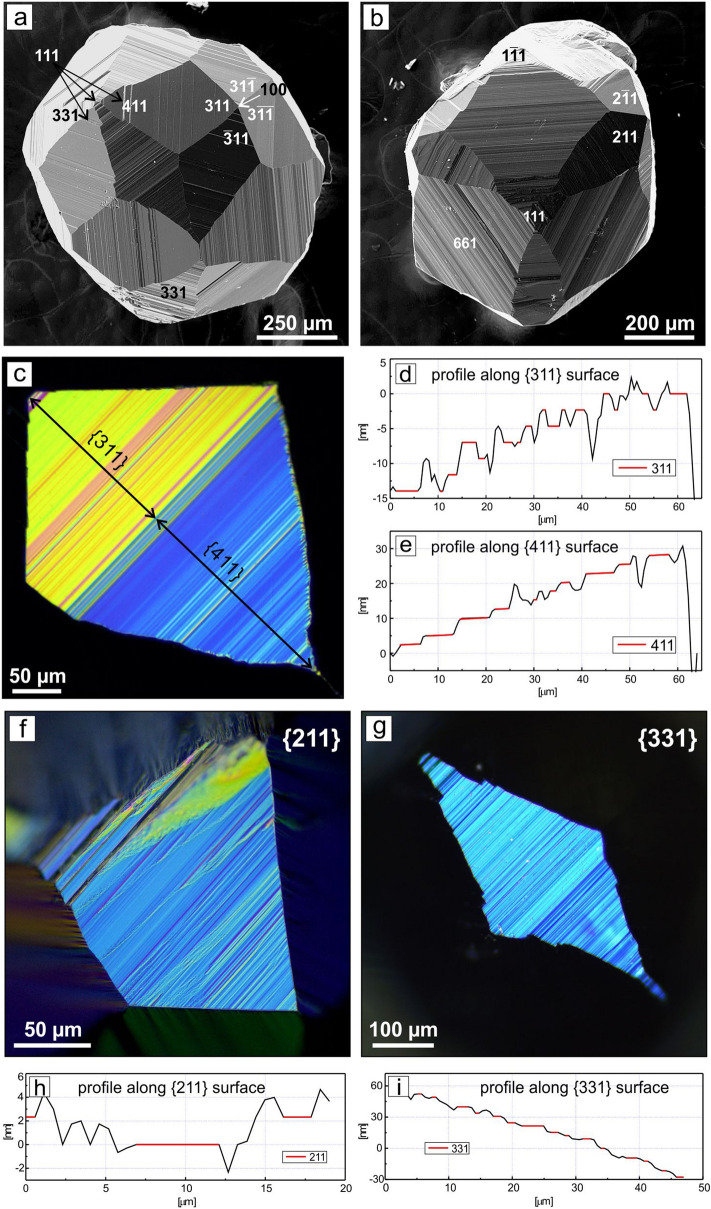
Morphology of combinational diamond crystals grown on the seeds. SEM micrographs of diamond crystals from (**a**) Er–C and (**b**) Lu–C systems; (**c**) DIC images of {311} and {411} faces and (**d, e**) profiles across these surfaces; (**f**) DIC image of the {211} face and (**h**) profile across it; (**g**) DIC image of the {331} face and (**i**) profile across it. Profiles were obtained using the TIC method.

The conditions and results of the experiments on diamond crystallization in the REM-C systems are presented in Supplementary Table S1. In the Sc–C system at 1900 °C, individual cube–octahedral diamond crystals were detected at the interface between the graphite capsule and the metal melt (Supplementary Fig. S1a). The crystals had black color and reached up to 100 μm in size. At the local areas of the seed crystals, insignificant growth was established. At 2000 °C, the starting graphite capsule converted to a diamond aggregate consisting of colorless and gray cube–octahedral crystals and blocks up to 1.5 mm in size. In the Y–C system, no diamond but metastable graphite was established at 1800 °C for 4 h. At 1900 °C, individual cube–octahedral diamond crystals (~ 80 μm) located at the graphite–metal interface were found. At 2000 °C, colorless, gray, and light brown cube–octahedral diamonds up to 500 μm in size were synthesized (Supplementary Fig. S1b). In all experiments with the Y–C system conducted at 1800, 1900 and 2000 °C, metastable graphite was found in the run products. In experiments with the La–C system at 1900–2100 °C, octahedral diamond crystals were synthesized (Fig. 1a) and slight diamond growth on the seeds was established. With increasing temperature, crystallized diamonds increased in their size from 50–100 μm to 250–300 μm. The color of the crystals changed from black to blueish gray. In the Ce–C system at 1900 °C, diamond in the form of small black polycrystalline aggregates showing elements of {111} faces was established in local areas at the graphite–metal interface. At 2000 °C, the degree of graphite conversion to diamond was about 70%. The produced diamonds were represented exclusively by octahedral crystals, their intergrowths and twins (Fig. 1b). Cyclic twins (Fig. 1c) were the most frequent. The maximum sizes of the crystals and blocks were up to 2 mm. Most diamond crystals were transparent and had blueish gray color. Praseodymium provided only crystallization of metastable graphite at 1900 °C. At 2000 °C, diamond growth on the seeds and synthesis of octahedral diamond crystals and intergrowths were established (Supplementary Fig. S1c). Diamond growth on the seeds took place with a rate of about 250 μm/h resulting in 1 mm sized octahedral crystals. In the Nd–C system, an insignificant growth was found on the {111} faces of the seed crystals in the range 1800–2000 °C. Single spontaneous diamond crystals up to 100 μm in size with {111} faces were established only at 2000 °C (Supplementary Fig. S1d). Metastable graphite was observed over the entire temperature range. In experiments with samarium, synthesis and growth of diamond was established at 1900 and 2000 °C. At 1900 °C, diamond crystallized in the form of individual aggregative crystals, and at 2000 °C, an aggregate of diamond crystals about 100 μm in size with a very high number of nucleation sites (~ 10^4^ cm^−2^) was formed at the graphite–metal interface. The crystals forming the aggregate showed octahedral morphology with minor cubic faces (Supplementary Fig. S1e). In the Eu–C system, neither diamond growth on the seeds nor spontaneous diamond synthesis was detected at temperatures 1900–2100 °C. In the Gd–C system, slight diamond growth in local parts of seed crystals was established in the temperature range 1800–2000 °C. Individual spontaneous diamond crystals were found only at 2000 °C (Fig. 1d). Terbium and dysprosium showed similar behavior as the catalysts for diamond synthesis. They provided diamond growth on the seeds and diamond spontaneous crystallization at temperatures 1800–2000 °C. The degree of graphite-to-diamond conversion in the Tb–C and Dy–C systems significantly depended on the temperature and increased from ˂˂ 1% at 1800 °C to 60% at 2000 °C. Diamonds crystallized from Tb and Dy catalysts had cube–octahedral morphology and showed mirror-smooth faces (Fig. 1e, Supplementary Fig. S1f). The color of the crystals varied from colorless to gray of various intensities. In experiments at 2000 °C, the crystals reached 0.6 mm in size. In the Ho–C system, diamond synthesis and growth on the seeds occurred at 1900 and 2000 °C (Fig. 1f). Diamonds crystallized at 1900 °C were bound by the faces of octahedron, cube and tetragon–trioctahedron. At 2000 °C, diamonds had cube–octahedral morphology and reached 0.7 mm in size. The crystals and crystal blocks were either colorless or gray colored. In experiments with erbium, spontaneous synthesis of diamond and diamond growth on the seeds was established at 1900 °C. At 2000 °C, diamond crystals with unusual shape and sizes up to 0.6 mm were synthesized. Their morphology was mainly determined by the stepped faces of tetragon–trioctahedron (Fig. 2a). The {100} and {111} faces had minor development or were absent at all.

Among the rare earth metals, thulium was found to belong to the group of the most effective solvent–catalysts. In the Tm–C system, diamond synthesis and growth was established in the temperature range 1800–2000 °C. The degree of graphite-to-diamond conversion sharply increased with increasing temperature and reached 80% at 2000 °C. The morphology of diamonds crystallized at 1900 °C was determined by the faces of octahedron, trigon–trioctahedron and rhombic dodecahedron (Supplementary Fig. S1g). At 1900 °C, the main forms of growth were the {111} and {hll} faces, and at 2000 °C—{111} and {100} faces. Diamond crystals were colorless and gray colored and reached 0.7 mm in size. In experiments with the Yb–C system, only diamond growth on the seeds was detected at 1900 °C. At 2000 °C, along with diamond growth on the seeds, spontaneous nucleation of diamond was observed. In all cases, the morphology of the crystallized diamonds was determined only by the {111} faces (Supplementary Fig. S1h).

In the Lu–C system, diamond synthesis and growth on the seeds were established in the temperature range 1900–2100 °C. At 1900 °C, a polycrystalline diamond aggregate formed at the graphite–metal interface; diamond growth on seeds was insignificant. The morphology of the crystals forming the aggregate was determined by the faces of the octahedron, trigon–trioctahedron and rhombic dodecahedron. At 2000 °C, the degree of graphite conversion to diamond was 90%. Diamond crystals and their fragments were mostly colorless and reached 1 mm in size (Fig. 2b). Crystal morphology was determined by the relative development of {111}, {hll}, {hkl} and {100} faces. With increasing temperature to 2100 °C the initial graphite capsule completely converted to a polycrystalline diamond aggregate, consisting of crystals, intergrowth, and fragments. The morphology of the diamonds was mainly determined by the {100} and {111} faces.

### General characteristics of diamond crystallization in the REM-C systems

The results presented in the preceding section demonstrate a contrasting behavior of the rare earth metals in their interaction with graphite at high pressures and temperatures and some general regularities. First of all, it should be noted that the nucleation of diamond in all cases occurs at the melt-graphite interface, and the growth of diamond proceeds via the FG (Film Growth) mechanism due to the carbon diffusion through the molten metal film. This mechanism has been described previously^21^ and found to be typical for various metallic and non-metallic solvent catalysts^6^. A characteristic feature of most systems studied in this work is a pronounced dependence of the degree of graphite-to-diamond conversion (α) on the temperature with a sharp increase in α at 2000 °C. The specific features of rare earth metals as solvents–catalysts for diamond synthesis are significantly different values of the degree of transformation of graphite to diamond and the appearance of metastable graphite. For example, in the Y–C and Nd–C systems, metastable graphite actively crystallized in the temperature range 1800–2000 °C, either along with diamond or without it. The appearance of metastable graphite is also found in the Pr–C, Gd–C, Er–C, Tm–C, and Yb–C systems.

**Figure 3:**
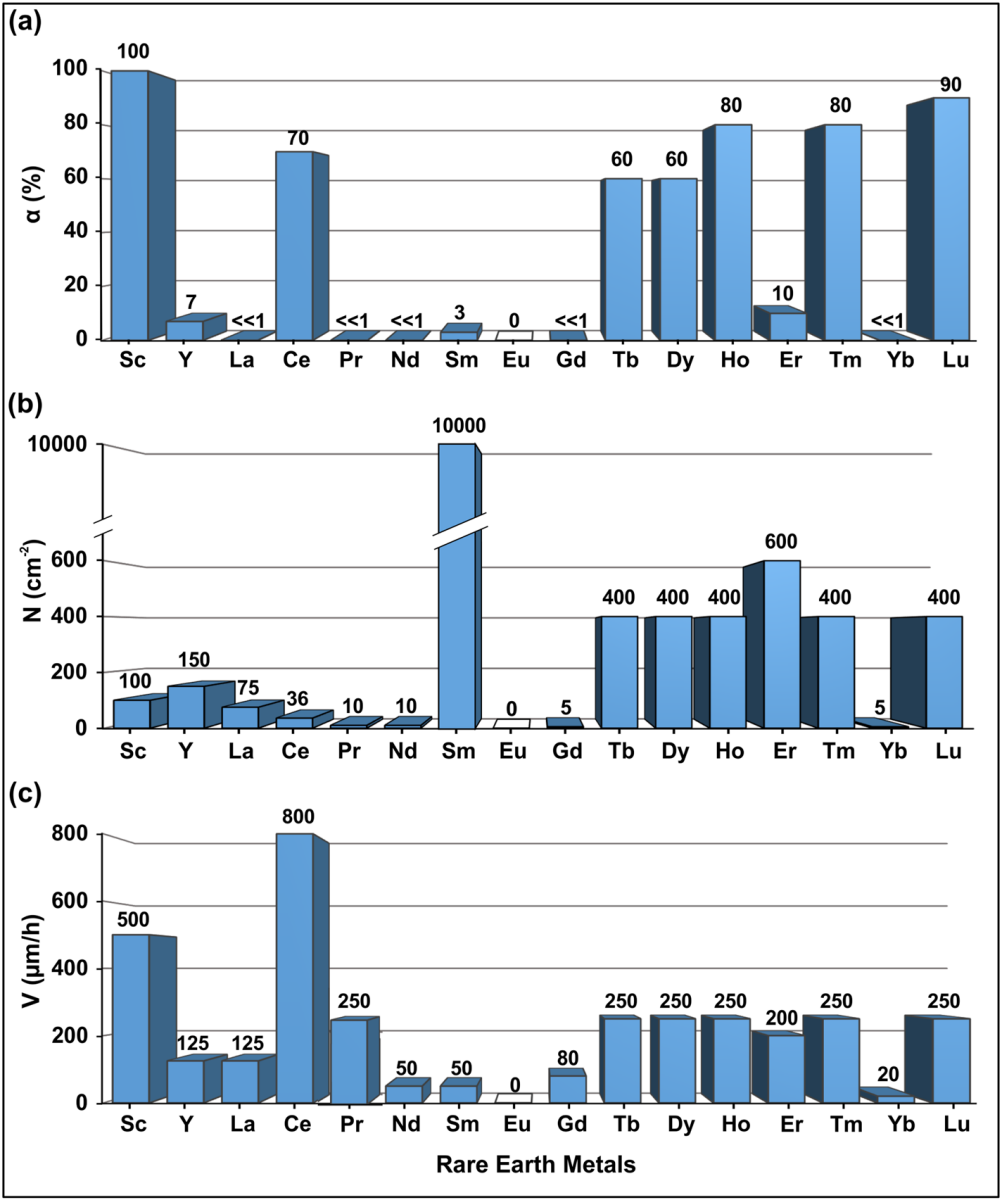
The results of diamond crystallization in REM-C systems at 7.8 GPa and 2000 °C. (**a**) Degree of graphite-to-diamond conversion; (**b**) density of diamond nucleation sites at the graphite–metal interface; (**c**) average linear diamond growth rates.

Figure 3 summarizes data on the degree of graphite-to-diamond conversion found in experiments at 2000 °C for 1 h, density of diamond nucleation sites at the graphite–metal interface and average linear growth rates. These results allow an integral assessment of the diamond-forming efficiency of different rare-earth metals in terms of diamond synthesis. The combination of relatively high growth rates from 125 to 800 µm/h with a relatively low densities of nucleation sites from 36 to 400 cm^−2^ leads to crystallization of relatively large (0.5–2 mm) diamond crystals in the melts of Sc, Y, La, Ce, Pr, Tb, Dy, Ho, Er, Tm and Lu. The most effective rare earth metals that provide maximum α values from 60 to 100% are Sc, Ce, Tb, Dy, Ho, Tm, and Lu. Diamond synthesis in the Sm–C system is peculiar since it is characterized by an abnormally large number of nucleation sites (~ 10^4^ cm^−2^) at low values of α and growth rates. The Eu–C system differs fundamentally from all other rare earth metals since only in this system neither synthesis nor growth of diamond is established due to the formation of europium carbide over the entire studied temperature range.

### Forms of diamond growth in the REM-C systems

The main forms of diamond growth in the REM-C systems are the {111} and {100} faces which are common for synthetic diamonds. It is well known that the relative development of these simple forms in the most studied transition metal melts changes from cube to octahedron with increasing synthesis temperature^5,22^. Such a dependence was not observed for most REM-C systems in the investigated temperature range (1800–2100 °C). The change in the morphology of diamond crystals with the synthesis temperature was established only for the Lu–C and Tm–C systems (Supplementary Table S1). In this case, crystal habit changed from octahedral to cube–octahedral with increasing temperature, while tetragon–trioctahedron and trigon–trioctahedron faces were observed for transitional forms.

Most pronounced is the dependence of diamond morphology on the composition of the REM solvent–catalyst (Supplementary Table S1). Cube–octahedral diamonds crystallize in the Sc–C, Y–C, Sm–C, Tb–C, and Dy–C systems. In the La–C, Ce–C, Pr–C, Nd–C, and Yb–C systems, diamond crystallizes exclusively in the form of octahedrons, irrespective of the temperature. When Gd, Dy, Ho, Er, Tm, and Lu are used as the catalysts, the tetragon–trioctahedra and trigon–trioctahedra faces are established on the crystals. The faces of these simple forms are, in most cases, stepped with combination hatching. According to goniometric measurements and TIC studies, sections corresponding to a series of faces of simple crystallographic indices are present on these surfaces (Fig. 2). The {311} faces are the most common and have significant development in the crystal habit. This simple form is also common for diamond crystals grown from melts of transition metals and copper^23–25^. To a lesser extent, {211}, {411}, {511}, {733} and {110} faces are observed for diamonds synthesized in the REM-C systems. In addition, the ends of the macrosteps on the {100} and {111} faces are often composed of surfaces corresponding to the faces {611}, {833}, {441}, {661} and {776}. In traditional metal–carbon systems with Fe, Ni, Co and Mn catalysts such a diversity of growth faces is not observed. Usually, the appearance of the {311} and {110} faces on diamond crystals in these systems is associated with a decrease in the growth rate, and the appearance the {511} or {711} faces is associated with the addition of nitrogen getters to the system^26–28^. These regularities do not apply to the morphology of diamond from the REM-C systems. As shown by the results of the study, diamond crystals with the additional faces grow at rates from 80 to 250 μm/h. At similar rates, depending on the composition of the REM-C system, crystals with octahedral and cube–octahedral habit without secondary faces can form (Supplementary Table S1). In addition, since all the diamonds studied belong to nitrogen free type II, the appearance of numerous additional faces on only some crystals cannot be associated with the getter properties of rare-earth metal melts.

Considering all the REM-C systems studied in this work, another interesting observation can be made. In the melts light rare earth metals (subgroup of cerium), crystallized diamonds have a simple shape composed only of (111) and (100) faces. On the contrary, diamond crystals synthesized from heavy REM (Gd–Lu) catalysts typically exhibit the faces of tetragon–trioctahedra and trigon–trioctahedra with both minor and major development. The exception is Tb and Yb in which cube–octahedral or octahedral crystals grow without secondary faces of other simple forms. At the same time, diamond crystals from the Er–C and Lu–C systems are polyhedrons composed of large combination surfaces {311}, {411} and {331} (Er) or {211} and {661} (Lu) (Fig. 2). The {111} and {100} faces are present as small facets that blunt the crystal vertices and form the ends of macrosteps on the combination surfaces.

### Spectroscopic characterization of diamonds synthesized in the REM-C systems

**Figure 4:**
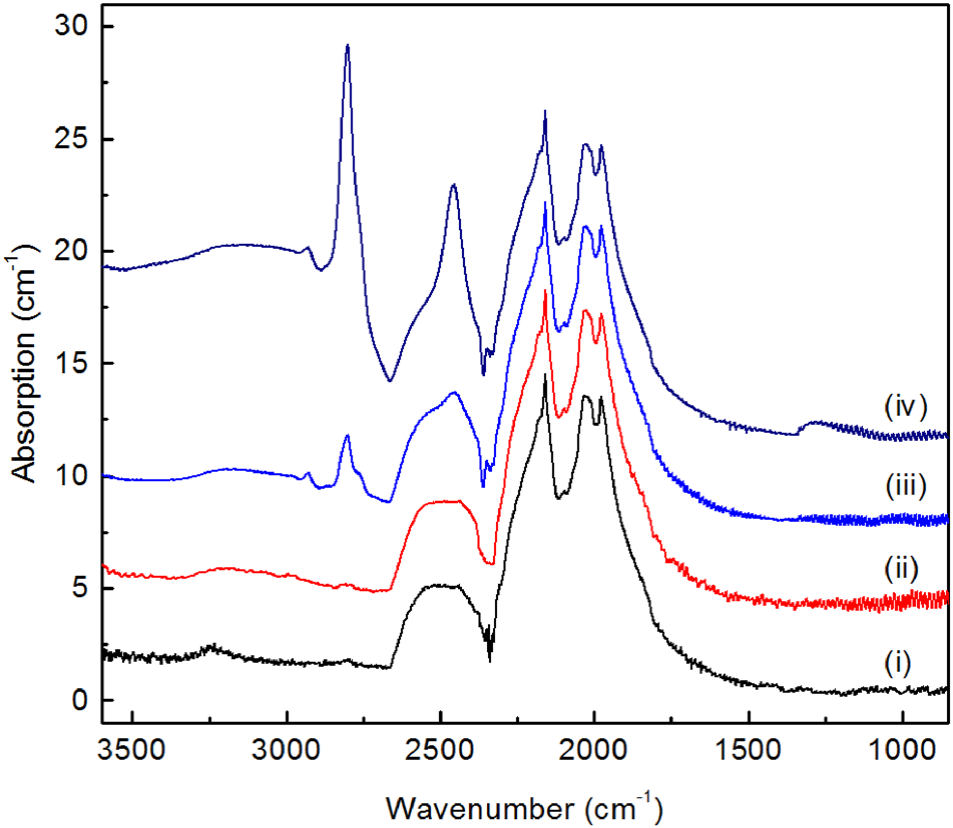
Representative infrared absorption spectra of diamonds produced in REM-C systems. The spectra were recorded for diamond crystals synthesized in (i) La–C, (ii) Ce–C, (iii) Tb–C and (iv) Lu–C systems at 2000 °C. The spectra were displaced vertically for clarity.

Diamond crystals synthesized from the rare-earth metal catalysts were studied by Fourier transform infrared (FTIR) absorption and photoluminescence spectroscopies. For the FTIR characterization, diamond samples with sizes and quality appropriate for absorption measurements were selected from the products of experiments conducted at 2000 °C. Of all rare-earth metal catalysts examined in this study, FTIR characterization was not possible for Eu, where no diamond synthesis was established, and for Nd and Gd, where synthesized diamonds had small sizes and poor quality. It is found that the produced diamonds show in the IR spectra either no impurity-related absorption, corresponding to type IIa diamond, or relatively weak absorption features related to boron impurities (type IIb). For all diamonds examined, no absorption bands due to nitrogen impurities were detected in the defect-induced one-phonon region of the spectra (Fig. 4). As a general tendency we found that diamonds produced from light rare-earth metals (La to Sm) preferentially corresponded to type IIa (Fig. 4i, ii), whereas diamonds from heavy rare-earth metals (Tb to Lu), as well as Sc and Y, more frequently showed B-related absorption in the IR spectra and corresponded to type IIb (Fig. 4iii, iv). The concentration of neutral substitutional boron in the studied diamonds was estimated from the strength of the absorption peak at 2800 cm^−1^ using commonly accepted calibrations^29^ and found to be within 0.1–1 atomic ppm. We suppose that the main source of boron in the growth system was the starting graphite containing trace amounts of B impurities.

Considering the observation that diamonds synthesized in the REM-C systems contain no nitrogen impurities detectable by IR absorption, it is worth noting that the traditional approach to grow nitrogen-free diamond from the conventional transition metal catalysts (e.g. Fe, Ni, Co) relies on the addition of elements that effectively react with nitrogen to form nitrides. These elements, termed “nitrogen getters”, typically include Ti, Al and Zr^4–6^. Recently, it has been demonstrated that silicon when added to a Ni-based metal catalyst may also act as the nitrogen getter presumably due to formation of Si_3_N_4_^30^. The rare-earth metals are known to have a strong affinity for nitrogen and the REM nitrides are very stable and comparable in stability to those of titanium or zirconium^31,32^. Therefore, it appears most likely that the crystallization of nitrogen-free type II diamond in the REM-C systems is enabled by the nitride forming ability of the rare-earth metal catalysts and their nitrogen gettering properties.

**Figure 5:**
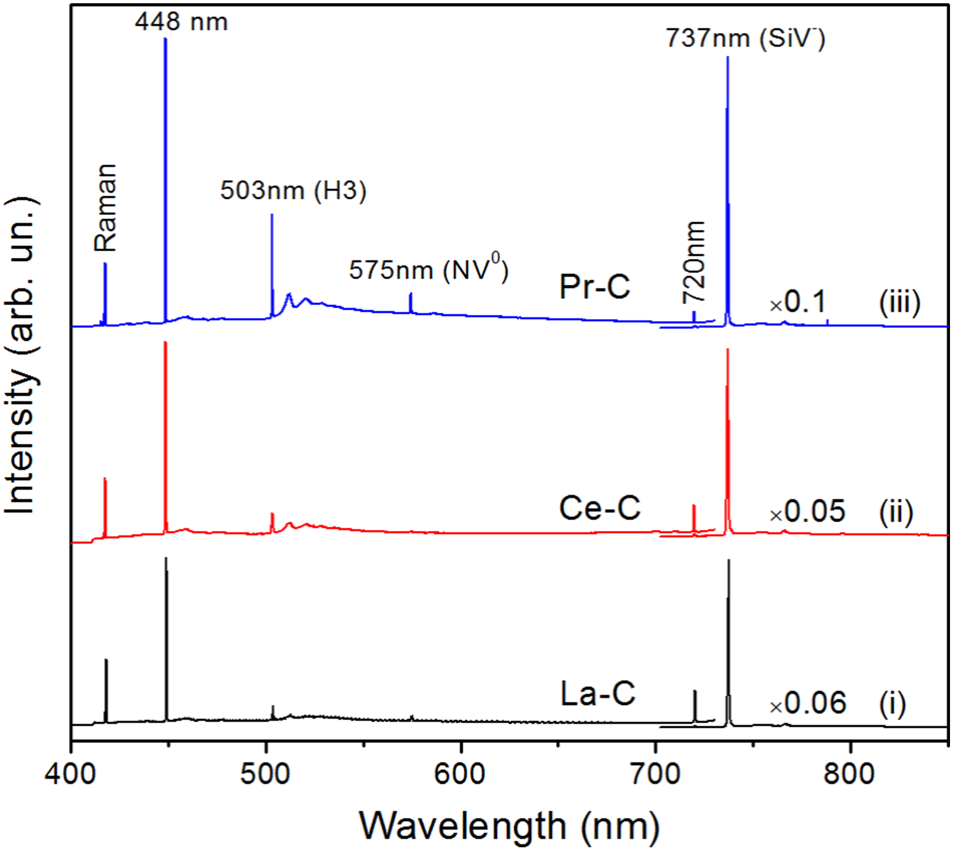
Representative PL spectra of diamonds produced using light rare-earth metal catalysts. The spectra were recorded at 80 K with excitation of 395 nm for diamonds synthesized in (i) La–C, (ii) Ce–C and (iii) Pr–C systems at 2000 °C. The spectra were normalized to show the same intensity of the diamond Raman peak and displaced vertically for clarity. The near-infrared parts of the spectra were multiplied by factors of (a) 0.06, (b) 0.05 and (c) 0.1.

Photoluminescence measurements were performed for diamond samples produced in the REM-C system at 2000 °C. It is found that irrespective of the rare-earth metal catalyst used in the experiments, the main PL features of the synthesized diamonds are related to negatively charged silicon-vacancy (SiV^−^) centers and a set of nitrogen-vacancy centers (NV, N_2_V and N_3_V). In addition, a few other PL systems were detected in the spectra, which will be considered below. Some general characteristics were observed for diamonds synthesized from light rare-earth metals (La, Ce, Pr, Sm) and heavy rare-earth metals (Gd–Lu). Figure 5 shows typical PL spectra recorded for diamonds from light REM catalysts. In this case, the spectra are dominated by the vibronic band with a zero-phonon line at 737 nm caused by the negatively charged silicon-vacancy centers (SiV^−^), nitrogen-vacancy centers are very weak and barely visible. As a rule, the intensity of the 737 nm line is 10–20 times higher than the intensity of the diamond Raman line, suggesting relatively high concentrations of the SiV centers in diamonds. In addition, a weak line at 720 nm was frequently present. An interesting feature found in the spectra of these diamonds is a narrow line peaking at 448 nm with the intensity typically exceeding that of the diamond Raman line. To the best of our knowledge, the 448 nm line has not been previously reported for synthetic diamonds. The nature of the defect responsible for the 448 nm peak is not clear at the moment and deserves further investigations.

**Figure 6:**
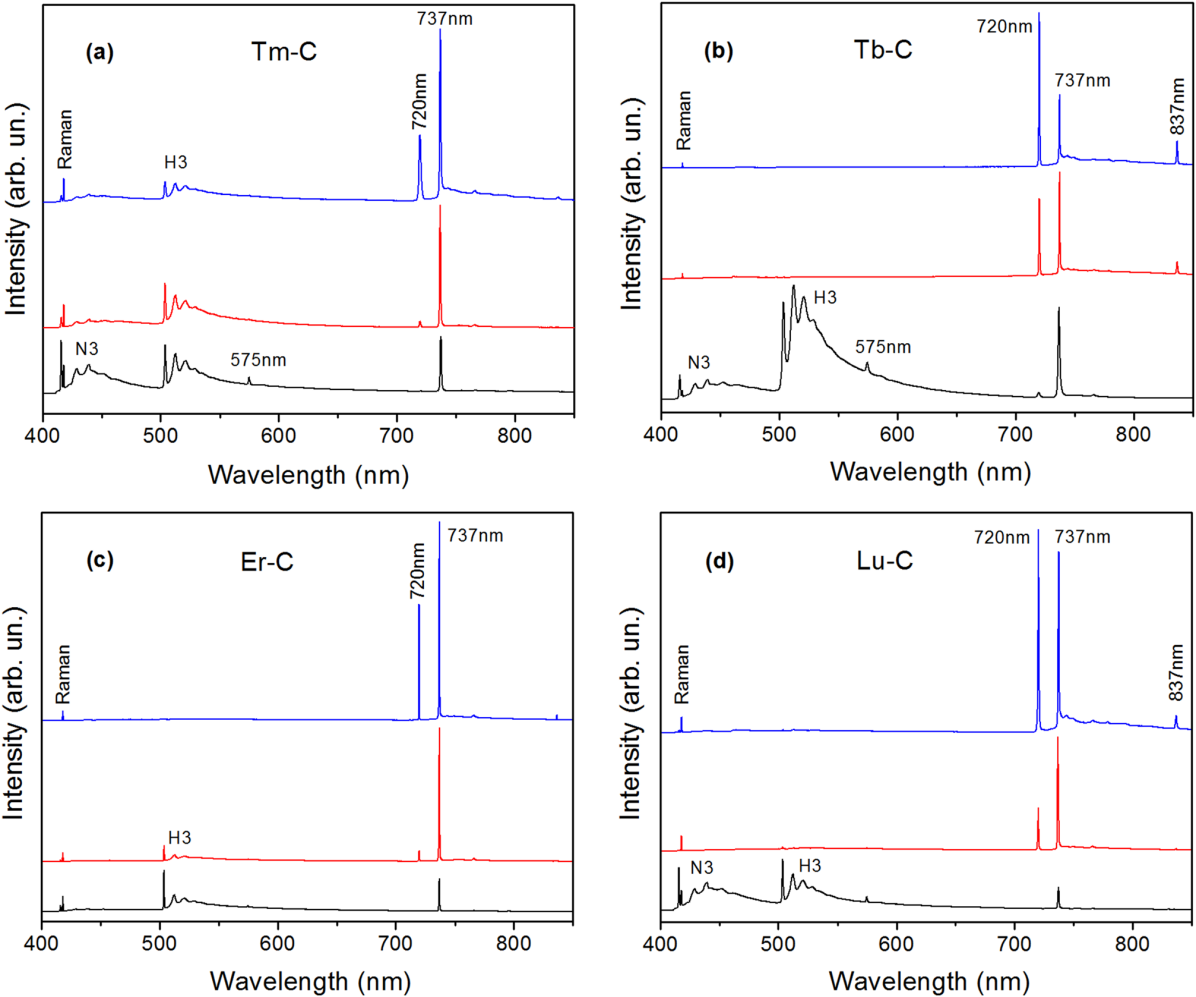
Representative PL spectra of diamonds produced using heavy rare earth catalysts. The spectra were recorded at 80 K with excitation of 395 nm for diamonds synthesized in (**a**) Tm–C, (**b**) Tb–C, (**c**) Er–C and (**d**) Lu–C systems at 2000 °C. The spectra in each panel were normalized to show the same intensity of the diamond Raman peak and displaced vertically for clarity.

Diamonds synthesized from heavy RE metal catalysts, as well as Sc and Y, showed more variable photoluminescence characteristics. In this case, diamond samples recovered from the same synthesis run demonstrated various PL spectra (Fig. 6), which can roughly be divided into three categories. First, there are samples where photoluminescence is dominated by the SiV centers with the other PL features being very weak. Another category comprises spectra where the dominant emission bands are related to the nitrogen-vacancy centers (H3 and N3). The SiV centers are also present in such spectra but with much lower intensities. Diamonds of the third category show specific spectra where the 737 nm line of the SiV centers is accompanied by an intense peak at 720 nm and a weaker peak at 837 nm. In some cases, the intensity of the 720 nm line exceeds that of the 737 nm line. It is interesting to note that no 448 nm line was detected in the PL spectra of diamonds synthesized from heavy rare-earth metal catalysts. The 720 nm line was previously observed in PL spectra of diamonds synthesized in the Mg–C and Mg–Si–C systems^13,33^. As a rule, its intensity was about an order of magnitude lower than that of the 737 nm line of the SiV centers. The reason why the 720 nm line attains relatively high intensities in diamonds produced in this study is not clear.

For the La–C, Er–C and Lu–C systems, we examined the effect of the crystallization temperature on PL characteristics of synthesized diamonds. It was found that diamond crystals produced at 1900 °C generally showed PL spectra similar to those produced at 2000 °C. Diamonds produced at 2100 °C as a rule exhibited higher PL intensities from the nitrogen-vacancy centers and lower PL intensities from the silicon-vacancy centers.

**Figure 7:**
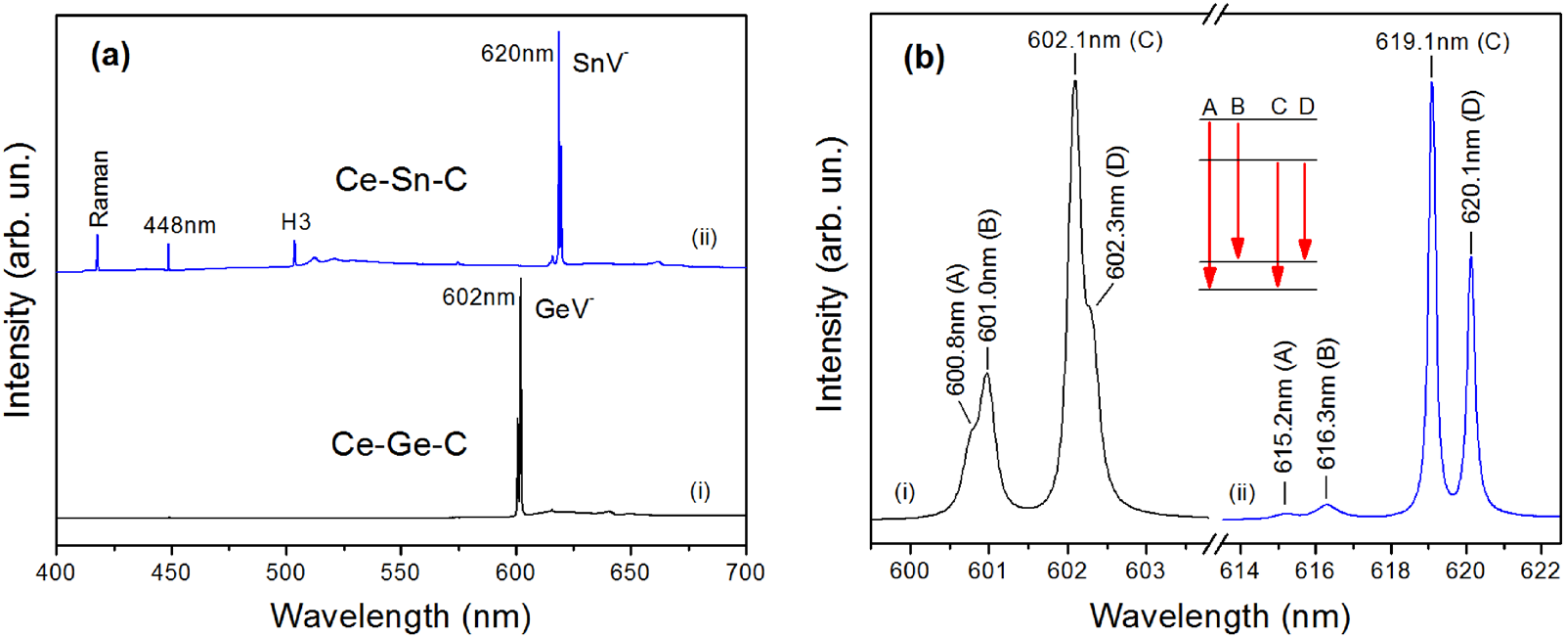
PL spectra of diamonds synthesized from the Ce catalyst with Ge and Sn additives. (**a**) Overview PL spectra of diamonds from (i) Ce–Ge–C and (ii) Ce–Sn–C systems. The spectra were recorded at 80 K with excitation of 395 nm and displaced vertically for clarity. (**b**) PL spectra in the region of zero-phonon lines of (i) GeV^−^ and (ii) SnV^−^ centers. The inset shows schematic structure of electronic transitions at the centers.

Our results demonstrate that the characteristic feature of the overwhelming majority of diamond samples examined in this work was the occurrence in the PL spectra of a vibronic system with zero-phonon line (ZPL) structure at 737 nm, corresponding to the well-known silicon-vacancy (SiV) centers. It should be noted that the main source of silicon impurities in the growth system was the starting graphite containing approximately 120 wt ppm Si^34^. The observed high efficiency of Si incorporation in diamonds synthesized in the REM-C systems has prompted us to examine the possibility of producing diamonds doped with other group IV elements (Ge, Sn) using the rare-earth metal catalysts. Several search experiments were performed in the Ce–C and Ho–C systems with the addition of 10 wt% of Ge and Sn. Photoluminescence characterization of the synthesized diamonds revealed that depending on the additive, the PL spectra were dominated by the emission bands with ZPL structures at 602 nm and 620 nm, corresponding to the negatively charged germanium-vacancy (GeV^−^) and tin-vacancy (SnV^−^) centers, respectively (Fig. 7). This finding proves successful doping of the diamonds with Ge and Sn and opens up an approach for producing diamond containing GeV and SnV optical centers.

## Conclusions

In summary, the catalytic ability of rare-earth metals for converting graphite to diamond at high-pressure high-temperature conditions has been experimentally established. The boundary conditions for diamond crystallization in the REM-C systems and the optimal parameters for single crystal diamond synthesis are determined. It is found that the degree of graphite-to-diamond conversion, density of diamond nucleation sites at the graphite–catalyst interface, and diamond growth rate depend on the REM solvent–catalyst and increase with temperature. The morphology of synthesized diamonds is controlled by the catalyst composition and does not depend on temperature. Along with diamonds showing octahedral and cube–octahedral morphologies, crystals with unusual habits built by tetragon–trioctahedron {311}, {411}, {211} faces and trigon–trioctahedron {311} and {661} faces are produced. Spectroscopic characterization reveals that diamonds synthesized in the REM-C systems contain no nitrogen impurities accessible by IR absorption spectroscopy and, therefore, correspond to type II diamond. This allows qualifying the rare-earth metals as both catalysts for diamond synthesis and nitrogen getters. Photoluminescence characteristics of the studied diamonds are determined by the well-known nitrogen-vacancy and silicon-vacancy centers along with a number of new currently unidentified optical centers. It is found that the REM catalysts provide growth conditions favorable for the incorporation of group IV elements (Si, Ge, Sn) in the diamond lattice with formation of SiV, GeV and SnV color centers. We believe that the approach developed in this study to synthesize diamond doped with group IV elements from the rare-earth metal catalysts can be useful for further investigations on the properties of the corresponding color centers and their possible applications in quantum technologies.

## Methods

Experiments in the REM-C system (REM = Sc, Y, La, Ce, Pr, Nd, Sm, Eu, Gd, Tb, Dy, Ho, Er, Tm, Yb, Lu) were carried out using a split-sphere multi-anvil high-pressure apparatus. The design of the employed high-pressure cells and the calibration data have been presented in our previous works^6,13,35^. All experiments were carried out at a pressure of 7.8 GPa in the temperature range from 1800 to 2100 °C. Experiments at 1900, 2000 and 2100 °C were run for 1 h and those at 1800 °C for 3 or 4 h. The starting materials were graphite rods (99.97% purity), rare earth metals (99.99% purity), and synthetic diamond seed crystals in the form of cuboctahedrons, 0.5 mm in size. The graphite rods were machined into thick-walled capsules (1.5 mm) with an outer diameter of 6.9 mm and a height of 6.5 mm. In the capsule center, a piece of a rare earth metal was placed in a hole 3.9 mm in diameter and 3.5 mm in height, and 4 seed crystals were put at the interface between the metal and graphite. The graphite capsules were enveloped from all sides with a 0.1 mm thick Mo foil to avoid penetration of the high-pressure cell components during experiments. The high-pressure cell assembly used in this study is demonstrated in Supplementary Fig. S2. To minimize REM oxidation, the assembled high pressure cells were dried in a vacuum oven at 100 °C for 24 h, followed by filling the oven with argon. In all experiments, the identity of the cell assembly and procedures for creating and maintaining the pressure and temperature were strictly controlled. For a comparative analysis of diamond synthesis efficiency in the REM-C systems, the degree of graphite conversion to diamond (α), which is defined as α = M_Dm_/(M_Dm_ + M_Gr_)  × 100, where M_Dm_ is the mass of the synthesized diamond and M_Gr_ is the mass of residual graphite, was determined for each experiment. In addition, the density of diamond nucleation sites at the graphite–melt interface and the average linear diamond growth rates were determined for experiments at 2000 °C for 1 h. After experiments, the products were dissolved in a hot mixture of nitric and hydrochloric acids. To remove graphite from the surface of the recovered diamonds, they were treated in a mixture of an aqueous solution of K_2_Cr_2_O_7_ and concentrated H_2_SO_4_. The morphology of diamond crystals was studied using optical and scanning electron microscopy. Goniometric measurements of diamond crystals were carried out on a GD-1 double-circle goniometer. The measurement accuracy of point light reflections from the faces was ± 2′. The microrelief of the faces was investigated by optical microscopy using a Carl Zeiss Axio Imager Z2m microscope. To increase the contrast of the face relief images, the method of differential interference contrast (DIC method) was used. The total interference contrast method (TIC method) was used to create profiles over the stepped combination surfaces and establish the presence of real faces on them. The profiles were created by scanning across the elongation of the hatching for the flattest sections of the combination surfaces. Scanning electron microscopy (SEM) studies of crystals were performed using a Tescan MIRA3 LMU microscope at the Analytical Center for Multielement and Isotope Research of the Siberian Branch of the Russian Academy of Sciences.

Spectroscopic characterization of synthesized diamond crystals was performed by means of infrared (IR) absorption and photoluminescence (PL). IR spectra were recorded using a Bruker Vertex 70 Fourier transform infrared (FTIR) spectrometer fitted with a Hyperion 2000 microscope. PL spectra were measured using a custom-built setup based on a Horiba JY iHR320 monochromator equipped with a Syncerity CCD detector. Photoluminescence was excited using a continuous-wave laser operating at 395 nm. A Linkam THMS350V heating/freezing stage mounted on an XYZ translation stage was used for the low-temperature measurements.

## Supplementary information

Supplementary Informations.

## Data Availability

All data generated or analyzed during this study are included in this published article (and its Supplementary Information files).
