# Supplementary material for: Rare-earth metal catalysts for high-pressure synthesis of rare diamonds
Source: Sci Rep. 2021 Apr 19;11:8421. doi: 10.1038/s41598-021-88038-5 (PMC8055970; doi:10.1038/s41598-021-88038-5)
Supplement: Supplementary file 1 — Supplementary Informations. [file 41598_2021_88038_MOESM1_ESM.pdf]

Supplementary Information

Rare-Earth Metal Catalysts for High-Pressure Synthesis of Rare Diamonds

Yuri N. Palyanov[1,2*], Yuri M. Borzdov[1], Igor N. Kupriyanov[1], Alexander F. Khohkhryakov[1,2], Denis V. Nechaev[1]

[1]*V.S. Sobolev Institute of Geology and Mineralogy Siberian Branch of the Russian Academy of Sciences, Academican Koptyug Ave., 3, Novosibirsk, 630090, Russian Federation.*

[2]*Novosibirsk State University, Pirogova str., 2, Novosibirsk, 630090, Russian Federation.*

*Correspondence to: palyanov@igm.nsc.ru.

Supplementary Table S1. Conditions and results of experiments on diamond crystallization in the REM-C systems.

Run No.	REM catalyst	T,°C	Time, h	DG	DN	α	Gr	Diamond morphology	N, cm^{-2}	V, μm/h
1168/7	Sc	2000	1	+	+	100	-	{100}, {111}	100	500
1164/7	Sc	1900	1	+	+	<<1	-	{100}, {111}		
615/8	Y	2000	1	+	+	7	+	{111}>{100}	150	125
1153/7	Y	1900	1	-	+	<<1	+	{111}, {100}		
1155/7	Y	1800	4	-	-	0	+			
1172/7	La	2100	1	+	+	<<1	-	{111}		
614/8	La	2000	1	+	+	<<1	-	{111}	75	125
1166/7	La	1900	1	+	+	<<1	-	{111}		
613/8	Ce	2000	1	+	+	70	-	{111}	36	800
1162/7	Ce	1900	1	+	+	<<1	-	{111}		
616/8	Pr	2000	1	+	+	<<1	-	{111}	10	250
1156/7	Pr	1900	1	-	-	0	+			
1171/1	Nd	2000	1	+	+	<<1	+	{111}	10	50
1154/7	Nd	1900	1	+	-	0	+	{111}		
596/8	Nd	1800	4	+	-	0	+	{111}		
1169/7	Sm	2000	1	+	+	3	-	{111}, {111}>{100}	10000	50
1165/7	Sm	1900	1	+	+	<<1	-	{111}, {100}		
1173/7	Eu	2100	1	-	-	0	-			
617/8	Eu	2000	1	-	-	0	-			
1167/7	Eu	1900	1	-	-	0	-			
590/8	Gd	2000	1	+	+	<<1	+	{111}, {100}, {311}, {411}	5	80
1150/7	Gd	1900	1	+	-	0	-	{100}, {111}, {311}, {411}		
591/8	Gd	1800	1	+	-	0	-	{111}, {100}, {311}, {411}		
1119/7	Tb	2000	1	+	+	60	-	{100}, {111}	400	250
1118/7	Tb	1900	1	+	+	3	-	{100}, {111}		
2089/2	Tb	1800	3	+	+	<<1	-	{100}, {111}		
1120/7	Dy	2000	1	+	+	60	-	{100}, {111}, {211}, {511}	400	250
1122/7	Dy	1900	1	+	+	3	-	{100}, {111}, {311}, {733}		
2090/2	Dy	1800	3	+	+	<<1	-	{100}, {111}, {411}		
1121/7	Ho	2000	1	+	+	80	-	{111}, {100}	400	250

590/8	Ho	1900	1	+	+	5	-	{111}, {311}, {100},		
592/8	Er	2000	1	+	+	10	+	{311}, {411}, {331}, {100}, {111}	600	200
1151/7	Er	1900	1	+	+	<<1	-	{100}, {111}, {311}		
1123/7	Tm	2000	1	+	+	80	-	{111}, {100}	400	250
592/8	Tm	1900	1	+	+	5	+	{111}, 311}		
1149/7H	Tm	1800	3	+	+	1	+	{111}, {311}, {110}		
1170/7	Yb	2000	1	+	+	<<1	+	{111}	5	20
1163/7	Yb	1900	1	+	-	0	+	{111}		
1152/7	Lu	2100	1	+	+	100	-	{100}, {111}		
1157/7	Lu	2000	1	+	+	90	-	{211}, {611}, {111}, {100}	400	250
1532/3H	Lu	1900	1	+	+	5-10	-	{111}, {211}, {110}		

"+" – was observed; "-" – was not observed; DG – diamond growth; DN – diamond nucleation; α – the degree of graphite-to-diamond conversion, $\alpha = (M_{Dm}/M_{Gr}+M_{Dm})\times 100$, where M_{Dm} is the mass of the synthesized diamond and M_{Gr} is the mass of residual graphite; N – the number of diamond nucleation sites at the graphite-catalyst interface per cm^2, V – the average linear diamond growth rate.

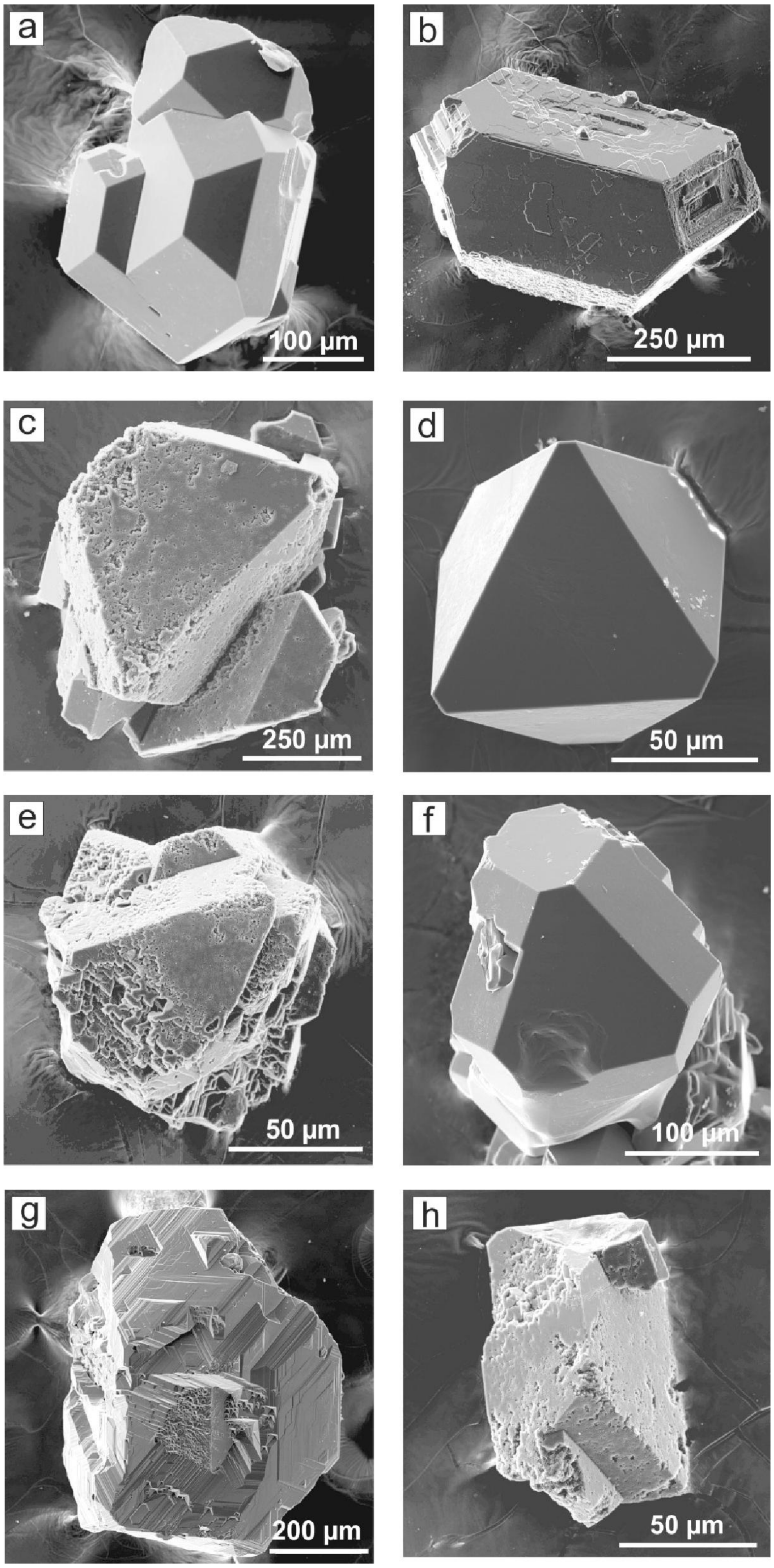

Supplementary Figure S1. SEM micrographs of diamond crystals synthesized in REM-C systems. (a) Intergrowth of cube-octahedral crystals, Sc-C system; (b) crystal of cube-octahedral habit, Y-C system; (c) intergrowth of octahedral crystals, Pr-C system; (d) octahedral crystal, Nd-C system; (e) intergrowth of octahedral crystals, Sm-C system; (f) cube-octahedral crystals, Tb-C system; (g) crystal of combinational form, Tm-C system; (h) octahedral crystal, Yb-C system.

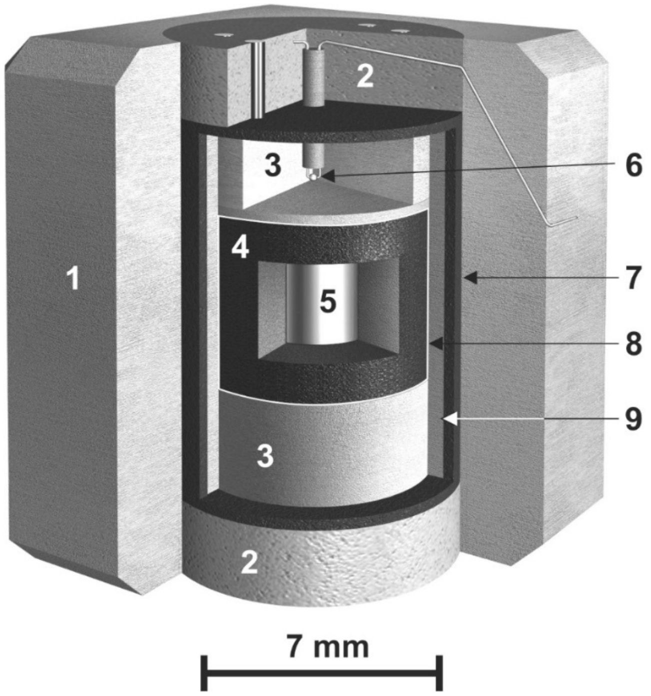

Supplementary Figure S2. High-pressure cell and sample assembly used for diamond synthesis. 1 – ZrO_2 ceramic container; 2 – talc ceramic; 3 – ZrO_2 ceramic; 4 – graphite capsule; 5 – rare-earth metal catalyst; 6 – thermocouple; 7 – graphite heater; 8 – molybdenum foil; 9 – MgO sleeve.
